# When Arne met J. L.: attitudes to scientific method in empirical semantics, ordinary language philosophy and linguistics

**DOI:** 10.1007/s11229-023-04106-5

**Published:** 2023-04-13

**Authors:** Siobhan Chapman

**Affiliations:** Department of English, University of Liverpool, Liverpool, UK

**Keywords:** Austin, J. L., Empirical semantics, Linguistics, Naess, Arne, Ordinary language philosophy, Scientific method

## Abstract

In the autumn of 1959, Arne Naess and J. L. Austin, both pioneers of empirical study in the philosophy of language, discussed their points of agreement and disagreement at a meeting in Oslo. This article considers the fragmentary record that has survived of that meeting, and investigates what light it can shed on the question of why the two philosophers apparently found so little common ground, given their shared commitment to the importance of data in the study of language. Naess and Austin held different views about two significant aspects of the relationship between scientific method and philosophical investigation. The first aspect concerns the nature of experimental data; Naess used the statistical analysis of data collected from non-philosophical informants while Austin advocated deliberation leading to agreement over usage by a few skilled experts. The second aspect relates to their respective attitudes to the role of theory in philosophical inquiry, attitudes which drew on discussions of scientific method, and its relevance to philosophy, from the early decades of the twentieth century. This article traces the evidence for these views on scientific method in Naess’s and Austin’s respective published work, and in the record of their Oslo meeting. It concludes with a brief overview of opinions about scientific method manifest in the decades since that meeting in various branches of linguistics. These opinions speak to the enduring importance of attitudes to scientific method in relation to our study and understanding of human language.

## Introduction

The focal point of this article is a meeting between two philosophers which took place in Oslo in the autumn of 1959. The only evidence for what was said at the meeting consists of a few pages of notes, written sometime after the event and published even later. The discussion seems to have been amicable enough, but it did not result in any significant philosophical breakthroughs or changes of opinion. The meeting is significant to the history of philosophy, nonetheless, because it was the occasion of a rare conversation between two of the main mid-twentieth century pioneers of empirical method in the philosophy of language. The philosophers in question were Arne Naess, the acknowledged leader of the school of Empirical Semantics, and J. L. Austin, who held a similar position in relation to ordinary language philosophy. Although the two men shared a commitment to the importance of data in the study of language, they differed both on the most suitable type of data to use and on the way it should be analysed: differences which surface in the brief record of their meeting in Oslo.

This article will address the question of why Naess and Austin disagreed at the Oslo meeting, given their shared commitment to an empirical basis for philosophical inquiry. In doing so, it will focus on two aspects of their respective views on the relationship between scientific method and philosophical investigation. The first concerns the nature of experimental data. Experiments were foundational to Naess’s philosophy, which was based on the use of large amounts of data collected by questionnaires or other experimental methods and analysed statistically. Austin was not necessarily opposed to philosophical experiments, and indeed at times he referred to his own work as ‘experimental’ (Austin, 1966, p. 429). But he had very different ideas from Naess about how philosophers should conduct experiments and gather data, arguing that the intuitions of one or a few skilled experts were sufficient. The second aspect of how Naess and Austin differed over scientific method relates to how they addressed questions concerning the role of theory in philosophical inquiry. These questions developed against a context of significant interest in the relationships between analytic philosophy and the nature of scientific theory in the early twentieth century, which is the subject of the next section. Evidence for Naess’s and Austin’s views on scientific method from their published writings will be reviewed in Sect. 3. Section 4 focuses on the meeting itself, and addresses the question of why, given Naess’s and Austin’s apparently shared commitment to the empirical study of language, it seems to have resulted in little real agreement. Subsequent empirical study of language is briefly surveyed in Sect. 5, concentrating on some relatively recent developments in various branches of linguistics that continue to develop the themes in Austin and Naess’s 1959 conversation.

## Analytic philosophy and scientific theory

In the 1930s, when Naess and Austin were beginning their philosophical careers, it would have been impossible for any young analytic philosopher to be unaware of questions concerning the relationship between philosophy and science. This was due in large part to the rise of logical positivism, which had challenged any assumption that philosophy could be a speculative or purely intuitive enterprise. The Vienna Circle had embraced logical positivism as an approach to philosophy which ‘faces modern times, rejects [metaphysics and theology] and takes its stand on the ground of empirical science’ (Carnap et al., 1929, p. 339). The class of philosophically salient statements comprised the propositions of formal logic, analytic statements guaranteed to be true by the rules of the language in which they were expressed, and observations about the physical world which could be verified by experience. All other purported statements, including ethical judgements and metaphysical speculations, were meaningless. They had no place in serious philosophical discussion; they were ‘pseudo-statements’.

Logical positivism was yoked, at least to begin with, to a commitment to inductive reasoning. Observation statements about particular experiences, such as ‘this raven is black’ and ‘that raven is black’, formed the basis of inductive generalisation, such as ‘all ravens are black’. What made such generalisations meaningful was the possibility of identifying the evidence on which they were based and, crucially, the types of future evidence which would serve to verify predictions which followed from them: predictions such as ‘the next raven I see will be black’. Logical positivism was therefore subject to the general ‘problem of induction’. Inductive generalisations can only ever aspire to degrees of probability; an inductive statement can never be judged conclusively true because it is always possible, however unlikely, that some future observation will fail to verify its predictions. The logical positivists had, in various ways, learnt to live with this problem. Carnap, for instance, had acknowledged that ‘definitive and final establishment of truth’ was not in fact possible for any synthetic sentence and had proposed that such sentences might be judged by degrees of ‘confirmation’ rather than by absolute ‘verification’ (Carnap, 1936, p. 420). But logical positivism still had its critics, including Popper, who proposed an entirely different way of judging statements.

Popper was interested in whether a statement was ‘scientific’ or not, rather than in whether it was meaningful. For Popper, the mark of a scientific statement was that it made predictions about future observations, and therefore that it was falsifiable. It was irrelevant how many observations might be made that were compatible with the statement; what was important was that it must be possible to give an account of what type of observation would demonstrate that it was false. It did not matter that a hypothesis could never conclusively be proved true; the hypothesis held as a statement of science until such time as evidence caused it to be abandoned or, more probably, modified to become progressively more successful. At least in his early work, Popper was apparently unconcerned about the initial process of hypothesis formation; the genesis of a scientific theory ‘may be of great interest to empirical psychology; but it is irrelevant to the logical analysis of scientific knowledge’ (Popper, 1935, p. 7). Science was concerned not with how a scientific theory was conceived but with whether it was possible to establish what would count as evidence against it; ‘it must be possible for an empirical scientific system to be refuted by experience’ (Popper, 1935, p. 18).

Popper was offering an outspoken new version of the hypothetico-deductive scientific method, and distinguishing it sharply from induction. In actual scientific practice, both then and now, data can of course be used both for exploratory purposes to produce tentative hypotheses and for confirmatory purposes to test those hypotheses. But for Popper even initial hypotheses are radically underspecified by the data. He stipulated a lecture in 1953 that the role of observation in the formation of a hypothesis is simply to stimulate the scientist ‘to operate with conjectures: to jump to conclusions—often after one single observation’ (Popper, 1957, p. 181). And even that single observation will be salient to the scientist because of a set of expectations that draw on pre-existing theories. What was at issue was the question not of what scientists should be doing, but of how the practice of scientists should be understood and described. For Popper: ‘Induction, i.e., inference based on many observations, is a myth’ (ibid.). It is simply not possible to advance knowledge on the basis of inductive generalisation, and attempts to do so, such as ‘Marx’s theory of history, Freud’s psycho-analysis, and Alfred Adler’s co-called “individual psychology”’ were pure speculations, not scientific theories (Popper, 1957, p. 156).

Popper’s polarising views and strident words notwithstanding, philosophers of Naess’s and Austin’s generation were under no obligation to come down firmly on one side or other of the opposition he drew between induction and hypothetico-deduction. But they were aware of differing views about verification, confirmation and falsification, and of the questions these raised as to whether data should drive theorising, or be used as a means of testing and improving theoretical explanations. Both data and scientific theory are explicitly addressed in the Oslo conversation.

## Empirical semantics and ordinary language philosophy

Born in 1912 and 1911, respectively, Arne Naess and J. L. Austin were both starting to develop their philosophical ideas during the heyday of logical positivism. Both were keen to establish a more solidly scientific basis for the study of language. However, they approached this from different personal and academic backgrounds.

Naess had first-hand experience of the operations of the Vienna Circle, having travelled to Austria as a young graduate in 1934 and attended some of their meetings. He saw philosophical issues as continuous with scientific ones and praised the logical positivists for denying ‘the existence of problems which cannot and shall not be treated as scientific problems, but as problems of a special, eternal and “higher” kind’ (Naess, 1938, p. 176). However, Naess argued that if something could be expressed in language it was worth serious philosophical attention, and therefore that dismissing many of the things that people ordinarily said as ‘meaningless’ because they were not verifiable was unjustified. He explained in an interview in the 1990s that the logical positivists assumed the right to comment on language without looking into the facts of usage ‘[s]o, to me, they were antiempirical, as they thought that their analysis of the use of “or”, for example, was much deeper than what you could get from statistics’ (Rothenberg, 1993, p. 28).

When Naess set about establishing his own method for philosophical investigation, he kept in mind this need for a properly empirical attention to language use. His first major piece of published research aimed to establish the nature of ‘the opinion of the ordinary man (the “non-philosopher”) on the notion of truth’ and to do so in a manner ‘satisfactory from a scientific point of view’ (Naess, 1938, p. 11). Naess argued that professional philosophers were too ready to comment on the nature of the ordinary conception of truth, and to use such comments to support their theories or to denigrate those of their opponents, without actually consulting any relevant evidence. His own intellectual background meant he was better placed than many of his philosophical contemporaries to put the discussion of the ordinary conception of truth on an empirical footing. After his visit to Austria he had spent some time in Berkeley, California conducting psychological research; he devised laboratory experiments to observe and record the behaviour of rats in mazes, and learnt how to analyse the results of these experiments statistically. Also in Berkeley he became interested in a new methodology being adopted in sociology: the use of questionnaires to obtain information about people’s opinions, and their accounts of their own behaviour (for more detail on Naess’s academic background see Chapman, 2008). In his study of the ordinary account of truth he drew on these methods, instigating questionnaire-based interviews with members of the public in order to prompt comments about the nature of truth, grouping these individual comments according to salient characteristics, and subjecting these groups to statistical analysis.

Naess concluded that it was simply wrong to base any argument on ‘the opinion of the ordinary man on the notion of truth’ because no such single opinion existed. People held many and disparate views on the nature of truth and, moreover, those views showed remarkable similarities to the major established philosophical positions on the subject. The data which Naess had gathered using questionnaires allowed him to reach these conclusions through a process of generalising from individual instances: of allowing the evidence to suggest scientific models which could then be tested out against further data. To be truly scientific, statements about the conception of truth, like any philosophical statements, should be capable of being reduced to ‘sentences about possible *observations* made or planned’ (Naess, 1938, p. 18, original emphasis); his conclusions were ‘inferences from the observational data’ (Naess, 1938, p. 20). Perhaps most strikingly, he compares these conclusions to everyday knowledge, in a passage which seems to acknowledge and accommodate the established problem of induction. His findings were necessarily based on the method of sampling and are therefore not ‘secure’ but this does not mean that they lack scientific value:

[O]ur daily-life-knowledge is a knowledge of correlations of the same type as those stated in this work, and so is much of our psychological and sociological knowledge: the difference is often only one of style: absolutistic formulations are very often predominant in fields where no earnest work is done and where careful statistical formulations are most needed.

(Naess, 1938, p. 20)

Soon after the publication of his study of the ordinary conception of truth, Naess was appointed to a chair in philosophy at the University of Oslo, and from this position he continued to build what became known as Empirical Semantics, extending his statistical method to other areas of linguistic investigation. For instance, he studied the philosophically loaded notion of synonymity, arguing that questions about whether words and expression share meanings could only be answered by gathering judgements about the possibility of substitution in specific contexts. He extended and refined his use of questionnaires, engaging non-philosophical subjects in laboratory-style experiments in which they were presented with controlled examples and questioned about permissible substitutions. Again, he emphasised the primacy of collected data; in Empirical Semantics ‘stress is laid upon observations of usage and the uncertainties as regards the outcome of such observations. What does “correct usage” mean operationally?’ (Naess, 1947/51, vol 1, pp. 5–6). Naess concluded that synonymity was not absolute but a matter of degree. Natural language was always and necessarily imprecise, since meaning was always dependent on contexts, speakers and hearers. However, that imprecision itself was available for empirical scrutiny and could be measured and compared. Forms of expression may be more or less precise, depending on how many interpretations empirical study finds to be possible. A relatively high degree of imprecision was often acceptable in everyday communication, but professional philosophers might need to go through a process of producing ‘precisations’ of the expression they use, in order to ensure that their intended meaning was clear.

Naess contemplated a new method to supplement his existing use of questionnaires and experiments. He was interested in the possibilities offered by direct observation of usage, for instance as represented in a large body of published text. The problems which beset ‘occurrence analysis’ were practical ones. Naess tried recording usages on index cards, producing numbered lists, and identifying the other words with which a target word typically occurred. But he was not satisfied that these methods gave him access to sufficient data for his purposes: ‘[t]he non-existence of a method by which meanings can be “seen” by observation of use is one of the strong reasons not to abandon the synonymity questionnaire’ (Naess, 1947/51, vol 6, p. 2). His proposal, for now at least, to balance the use of occurrence analysis and questionnaires again points towards a commitment to scientific statements built up on the basis of available evidence and then tested out against further relevant data. Often attempting occurrence analysis based on single texts demonstrates that:

There is insufficient material to confirm or disconfirm strongly any hypothesis of interest. One way out of the difficulty is to create a supplementary text of high relevancy. This can be done by questionnaire methods. The questions can be formed in such a way that answers are apt to throw light on just those hypotheses which are tentatively formed on the basis of occurrence analysis. Generally, however, it is convenient to use questionnaires at first, and then go into occurrence analysis, or to mix both methods during all the stages of the investigation.

(Naess, 1947/51, vol 6, p. 52)

In his writings throughout the rest of the 1950s, Naess remained committed to the primacy of data, collected or observed. He commented ruefully on the fact that ‘the training of graduate students of philosophy seldom includes empirical research techniques and this further increases their already strong tendency to find systematic observation and step-by-step generalization from obtained data, irrelevant or unenlightening’ (Naess, 1956, p. 8). He starts an article on the use of questionnaires to investigate synonymity, published just after his meeting with Austin, with a highly enthusiastic account of his experience as a data-driven scientist:

If carried out with an eager and open mind, painstaking empirical research leads us into vast unchartered regions of facts and relations. The more one penetrates into the thickness of such regions, the more one is fascinated. One is—often against ones will—drawn further and further into the study of details and intricate structures revealed by the data found or collected.

(Naess, 1960, p. 481)

Austin would have applauded Naess’s emphasis on the benefits of ‘painstaking empirical research’. He was convinced that empirical research was the key to the successful study of language, and that it was indeed a slow and meticulous process. In the 1930s he too reacted against what he saw as unempirical tendencies in the accounts of language such as that offered by the logical positivists. But unlike Naess, Austin had no background or experience in the experimental sciences. In a rough series of notes not intended for publication, he commented on himself and his fellow Oxford philosophers: ‘remember all brought up on classics: no quarrel with maths etc., just ignorant’ (Urmson et al., 1969, p. 83). Instead, as a student of Classics he had developed a sensitivity to the use of natural language and also something approaching a reverence for its powers of expression and discrimination. When he wanted to confront what he saw as logical positivism’s unempirical dismissal of many of the types of statements made in everyday communication as ‘meaningless’, he called on these resources. For Austin, trained and skilled speakers of a language had at their disposal ready-made knowledge of the full range of ways in which that language could and could not be used.

Austin’s reflections on the subtleties of usage led him to reject any simplistic account of language in terms of a one-to-one relationship between words and meanings. There was, he argued, ‘no simple and handy appendage of a word called “the meaning of (the word) ‘x’”’ (Austin, 1940, p. 30). It was always necessary carefully to consider patterns of acceptability and co-occurrence before making any pronouncement about meaning. This technique is apparent in some of the work that Austin was engaged in during the 1950s. In his 1957 Presidential address to the Aristotelian Society, ‘A plea for excuses’, for instance, he tackled issues surrounding the attribution of blame and responsibility. Posing the question of when actions could appropriately be described as ‘voluntary’ or ‘involuntary’, he proposed to proceed ‘by examining *what we should say when*, and so why and what we should mean by it’ (Austin, 1957, p. 7, original emphasis). Austin’s investigation led him to conclude that it is often simply not possible to apply either an adverbial or its apparent antonym. By their nature most descriptions are not amenable to modification; ‘[t]he natural economy of language dictates that for the *standard* case covered by any normal verb,—not, perhaps, a verb of omen such as “murder,” but a verb like “eat” or “kick” or “croquet”—no modifying expression is required, or even permissible’ (Austin, 1957, pp. 15–16). Austin illustrates this point with the following scenario: ‘[i]t is bedtime, I am alone, I yawn: but I do not yawn involuntarily (or voluntarily!), nor yet deliberately. To yawn in any such peculiar way is just not to yawn’ (Austin, 1957, p. 16).

For Austin, information about ‘what we should say when’ was the essential starting point in the philosophy of language. But unlike Naess, he was not of the opinion that such information was best or most reliable if it was collected by mass observation or experiment. In the published version of a lecture given in 1958 in Chicago, he proposes to start with cases:

Actual cases would of course be excellent: we might observe what words have actually been used by commentators on real incidents, or by narrators of fictitious incidents. However, we do not have the time or space to do that here. We must instead imagine some cases (imagine them carefully and in detail and comprehensively) and try to reach agreement upon what we should in fact say concerning them. If we can reach this agreement, we shall have some *data* (‘experimental’ data, in fact) which we can then go on to *explain*.

(Austin, 1966, p. 429, original emphases)

The emphasis on working ‘carefully’ and ‘comprehensively’ was of crucial importance to Austin. Those trained in the complexities of language, working systematically towards finding agreement on a particular linguistic issue, were likely to produce the most reliable data: data which could justifiably be called ‘experimental’. He encouraged collaborative collection of such data at his ‘Saturday Mornings’: meetings he arranged throughout the 1950s for a group of like-minded philosophers. Austin and his colleagues would work together to build up a picture of the ways in which the language was used to describe a particular area of experience, drawing on words used to define each other in dictionaries, and trying out different contexts and invented scenarios in which they could and could not appropriately be applied. With this technique, Austin ‘thought that he had devised a sort of “laboratory technique” which could be fruitfully used for finding solutions’ to philosophical problems (Urmson et al., 1969, p. 77).

Austin differed from Naess, then, not in his interest in putting the study of language on to an empirical and even a scientific footing, but in his views on the nature and origin of the information that offered the most reliable ‘experimental data’ for that study. The two differed in another significant way, too. In his early work, Naess argued that philosophical statements should be developments of ‘observations made or planned’, and he appears to have upheld this doctrine throughout his work on language. In articles such as ‘A plea for excuses’, Austin also proposed to build up a general picture about language use from looking at accumulated examples. But in other work he demonstrated a readiness to introduce theoretical entities into his account of language. His work on speech acts, presented at his William James lectures in Harvard in 1955, and later published as *How to do Things with Words* posits the existence of elements in the linguistic system which are not available to empirical observation, but can offer a systematic explanation of how language is used. In this, also, he was rejecting what he saw as the inappropriate emphasis on what he described in scare quotes as ‘“the meanings of words”’ (Austin, 1962, p. 100). Uttering a particular set of words with particular meanings was a significant aspect of language use, but it was only one aspect. What Austin labelled the ‘locutionary act’ needed to be considered alongside the ‘illocutionary act’ (determined by ‘in what way we are using the locution’, Austin, 1962, p. 98) and the ‘perlocutionary act’ (the ‘consequential effects’ of saying something, Austin, 1962, p. 101) in order fully to establish what speech act had been performed. As Harris and Unnsteinsson have observed, the distinction between the locutionary, the illocutionary and the perlocutionary reveal a side to Austin’s thinking that was ‘armed with systematic distinctions and stripped of an aversion to positive theorizing’ (Harris & Unnsteinsson, 2018, p. 387).

Some of Austin’s philosophical contemporaries identified the theoretical elements in his work on speech acts as a weakness, precisely because they introduced unobservable entities. In a paper originally published in 1963, Black complained that ‘The only proper unit for investigation seems to be what Austin has called an illocutionary act and the supposed locutionary act is at best a dubious abstraction’ (Black, 1969, p. 410). For Cohen, on the other hand, it was precisely the illocutionary act which was the problem, and he argued against ‘any attempt to prise off this aspect of meaning, and regard it not as meaning but as something else’ (Cohen, 1969, p 429). Grice was critical not so much of Austin’s attempts to introduce theoretical elements into his account of language, but of how he did it. Late in his life he informally voiced the opinion that, while Austin was far from being averse to theorising, ‘he didn’t do it very well, because he didn’t know what was required.^1^

Despite the opposition or the scepticism of some of his colleagues, Austin’s introduction of abstract, theoretical elements into his philosophy of language had resonances with what was happening across the Atlantic in the developing new field of theoretical linguistics. In the autumn of 1959, Austin’s ‘Saturday Mornings’ were devoted to a painstaking reading of Chomsky’s *Syntactic Structures*, which had been published a couple of years earlier (Warnock, 1973, p. 36). Austin had previously met Chomsky in Harvard in 1955, and Longworth suggests that: ‘it may be that it was his meeting with Chomsky that induced him to consider whether the study of language that he envisaged might ultimately fall within the purview of science, rather than philosophy’ (Longworth, 2020, p. 145). There is evidence that Austin was particularly impressed by Chomsky’s ambition to bring order and precision to the immensely complex domain of the grammar of natural language (see Chapman, 2005, p. 86 for an account of Grice’s report of this response). In *Syntactic Structures*, he would have found a concrete account of how that might work in practice; Chomsky reimagined grammar not as a descriptive account of a language but as ‘essentially a theory’ of the language, able to generate all and only the sentences which a native speaker would recognise (Chomsky, 1957, p. 49).

Chomsky implicitly dismissed the possibility of progress via inductive generalisation from observation; ‘the set of grammatical sentences cannot be identified with any particular corpus of utterances obtained by the linguist in his fieldwork’ (Chomsky, 1957, p. 15). The production of a grammar was not the linguist’s main or most challenging task. In fact, Chomsky was *nonchalant* as to how the linguist might construct the grammar, much as Popper was open to the possibility of all sorts of procedures for producing an initial scientific statement: ‘one may arrive at a grammar by intuition, guesswork, all sorts of partial methodological hints, reliance on past experience etc’ (Chomsky, 1957, p. 56). What was important was to be able to establish an objective way in which the grammar could subsequently be evaluated, modified or even abandoned. In later work, Chomsky asserted that: ‘It is quite certain that serious hypotheses concerning a native speaker’s knowledge of English, or concerning the essential properties of human language … will “go beyond the evidence”, if they did not, they would be without interest’ (Chomsky, 1969, p. 66). In Chomsky’s readiness to ‘go beyond the evidence’, Austin may have found endorsement of what he was doing in some of his own work on language.

## The meeting

By the late 1950s, Naess and Austin were both prominent advocates of empirical methods in the study of language and each headed up a recognised philosophical movement or school. It was perhaps inevitable that they should be curious about what they had in common, and about what separated them, and that they should eventually meet to discuss their similarities and differences. It is not clear exactly when or how Austin first learned about Empirical Semantics, but he seems to have been well aware of it by 1956. Warnock later reported Austin as returning from a visit to America in that year ‘a good deal perturbed by what he thought to be the increasing prestige there of Arne Naess’ (Warnock, 1973, p. 43). According to Warnock, Austin agreed with the general ambition for an objective and meticulous approach to philosophical progress, but was worried that Naess and his team were going about it the wrong way. At the same time, Naess was finding out about Austin’s ideas from his published work. In 1957 he was with a group of fellow Empirical Semanticists at the University of California, Berkeley, conducting experimental work which included studies designed to scrutinise Austin’s conclusions in, for instance ‘A plea for excuses’ (Murphy, 2014, p. 346).

Austin made a trip to Berkeley in 1958, while Naess was still there as a visiting professor. Given their respective reservations, it is no surprise that when they finally met they appear to have found little common ground. Warnock reports Austin as distinguishing carefully between his approach and what he, or perhaps Warnock on his behalf, described as ‘the kind of Gallup-poll, empirical team-work which Neass believed in, and which Austin regarded as, in principle, misguided’ (Warnock, 1969, p. 14n). Tantalisingly, it seems that there is, or once was, some sort of record of this first meeting, but Warnock describes it as ‘neither perfectly clear nor certainly reliable’ (ibid.). Happily, there is a more trustworthy, indeed a first-hand account of what took place during Naess and Austin’s second and final meeting. In the autumn of 1959 Naess was back in Oslo and Austin was visiting and giving some lectures. There were no doubt many conversations and discussions during Austin’s trip to Oslo, but the one from which a written record survives relates to a lecture in which Austin had discussed an article published the same year: ‘What should we say?’ by Herman Tennessen.

Naess was very much on home territory on this occasion, and not just physically. Tennessen had been Naess’s student and then colleague and had joined the expedition to Berkeley in 1957 (unlike Naess’s visiting professorship, Tennessen’s post was permanent and he had stayed behind when Naess returned to Oslo). Further, ‘What should we say?’ was published in *Inquiry*, the journal which Naess had founded in 1958 and which he still co-edited. It reported on empirical studies which Tennessen and his team had recently conducted in Berkeley, in which they sought responses from informants, from pre-school age through to adult, about what they believed they could or could not say in various contexts. Tennessen’s basic tenet, set out at the start and then borne out in his empirical findings, is that: ‘Any transmitter (e.g., any sentence) can transmit any message (e.g., any statement)’ (Tennessen, 1959, p. 266). That is, there are no binding rules, either of language or of usage, that determine what words mean; any use of language can potentially convey any meaning, depending on the context.

Tennessen employs the Empirical Semantic notion of precization to support his reasoning. People, perhaps particularly children, may be inclined to stipulate that there are various things that you ‘can’ and ‘cannot’ say. In answer to the question ‘Can you call a dog a cow?’ Tennessen’s young informants were very likely to answer in the negative. But on investigation such judgements were found to depend on one type of precization of the question, which can be represented as ‘is it permissible (correct, in place, not silly, etc.) to utter (write)…’ (Tennessen, 1959, p. 268). There is an alternative, equally possible precization along the lines of: ‘is it (technically etc.) within our power to utter (write)…’. Once that precization was activated, the children were much more likely to give a positive response. Similar experiments revealed equally fluid linguistic intuitions in the older age groups.

In his next experiment, Tenessen moved on directly to addressing Austin’s claims in ‘A plea for excuses’; in effect, he presented his test subjects with questions about ‘what we should say when’. In this experiment: ‘198 adult respondents (not students)’ were presented with eighteen ‘Should we ever say…’ questions and were asked both to answer with ‘yes’ or ‘no’ and also to ‘state their *reasons why we should say*…, and *why we should not say*… respectively’ (Tennessen, 1959, p. 279, original emphasis). The examples Tennessen’s subjects were presented with include the question of whether we should ever say ‘I yawn voluntarily’ or ‘I yawn involuntarily’. Tennessen was particularly interested in reasons given for negative answers to these questions, in which his subjects showed considerable agreement. Many respondents reported that the classification of certain actions as voluntary or involuntary was too obvious; they would not say it because it was trivial or redundant. Tennessen concludes that: ‘It must be this well-known phenomenon which has led some philosophers to believe that we would (should? can?) only say “x is voluntary” provided something seems fishy about x’ (Tennessen, 1959, p. 284; Tennessen was here alluding to Stanely Cavell, who had used the term ‘fishy’ in this context in his 1958 defence of Austin). Tennessen’s next step is to demonstrate that there are in fact contexts, however unusual, in which we might explicitly state that an obviously voluntary action is voluntary. If trying to decide on a borderline or ‘fishy’ case, it might be advisable to agree on an obviously voluntary action and to say, for instance, ‘I go to enjoy a good movie voluntarily’, so as to be able to compare this with the more controversial action. ‘Thus’, Tennessen concludes, ‘there *are* cases where it is necessary to call a spade “a spade” and a voluntary action “a voluntary action”’ (Tennessen, 1959, p. 284, original emphasis). It is never possible to make definitive statements about what we can or should say because this is always dependent on context and purpose in individual acts of communication.

The written account of the Oslo meeting was later published under the joint authorship of Austin and Naess, with a note describing it as ‘based on lecture notes taken during John Austin’s stay in Oslo in the autumn of 1959—with some further reflections by Arne Naess’. There is no attempt at verbatim accuracy; Naess offers ‘some hunches about Austin’s criticisms, and some tentative formulations of them’ (Austin & Naess, 1964, p. 144). The account begins with a reconstructed dialogue, with turns taken by ‘Austin’ and by ‘A.N.’, but it then continues in connected prose. Nevertheless, it is possible to establish something of what Austin and Naess each said.

Austin ‘had many good things to say about Tennessen’s article’ (Austin & Naess, 1964, p. 144). These are not specified—the loose transcription leads straight into a discussion of Austin’s objections—but it is not hard to identify aspects of ‘What should we say?’ that would have appealed to him. Although Tennessen used different terminology in which to express himself, Austin would have agreed with his rejection of any fixed relationship between ‘transmitter’ and ‘message’; for Austin too, the meaning of a sentence did not determine what statements (or other illocutionary acts) it could be used to perform. But Austin was never going to agree with Tennessen about the possibility of routinely saying ‘He yawned voluntarily’. In refuting Tennessen’s empirical findings, Austin was quite ready to blame the informants; they ‘gave wrong answers concerning their own use’ of expressions, perhaps because of lack of familiarity with the word ‘voluntarily’ itself; ‘[i]t is too difficult a word, maybe’ (Austin & Naess, 1964, p. 144). Austin stuck by his general claim that ‘adverbs are only used in exceptional cases, not standard cases (situations)’ and that this was a fact about the language system itself, because to say that a certain form of words, x, is never said, is equivalent to saying ‘there is a rule against saying x’ (ibid.).

It is significant that Austin was prepared to specify what is and is not said, and therefore what is and is not possible in the language system, not just in the absence of support from Tennessen’s experimental subjects, but in fact in direct opposition to their intuitions. This brings out one of the reasons why Naess and Austin disagreed despite apparently having so much in common. Austin was not against the involvement of non-philosophers in the collection of data; he had conceded that ‘actual cases’ of language use could be useful. What he was not prepared to accept were the intuitive responses of non-philosophers to questions of what they would or would not say. Such subjects were likely to make mistakes, or to reach faulty judgements about their own usage. If intuitive responses were to be of any value, specialist training in and sensitivity to language use was necessary. As Longworth puts it, ‘Austin clearly felt that his competence with “voluntary”’, as honed by reflective training, put him in a position to correct naïve opinion’ (Longworth, 2018, p. 951). Warnock had reported Austin as seeing the ‘Gallup-poll’ style methods of empirical semantics not just as unnecessary or as impractical but actually as ‘misguided’.

The Oslo discussion moves on to deal more explicitly with the competing merits of experimental and non-experimental methods. Austin had previously used the term ‘experimental’ to describe his own collaborative work with his colleagues, but here he seems to restrict it to type of methodology employed by Tennessen and by Naess. He is not inclined to use such methods himself; rather ‘Austin is going to use tapes in order to get first class observational material, stressing the oral character of language’ (Austin & Naess, 1964, p. 146). Austin’s own informal, unpublished notes mention that he had ‘no quarrel with maths etc’, and a similar attitude emerges in his discussion with Naess. It is, quite simply, difficult to design experiments; ‘one should not impose this technique upon [those] who have neither training in or aptitude for any experimental techniques or empirical approach’ (Austin & Naess, 1964, p. 147). Austin also points towards what he sees as a potential danger: ‘The success of particular empirical techniques within certain fields has had a curious anti-empirical effect; uncritical expectations that the use of the techniques in new fields will give better results than non-formal, intuitional, ways of confrontation with so-called “facts” or “data”’ (ibid.). The suggestion that assumptions about the superiority of experimental methods could in fact be detrimental to advancement in the study of language was a striking one for Austin to make in Oslo in 1959, but it was one that was to resurface decades later in relation to semantics, as will be discussed in the next section.

Towards the end of the record of the conversation, Naess proposes a summary of a type of activity which:

…may not be far away, for example, from the interpretation of J. L. Austin of hiw [sic] own activity or rather of the wider frame-woek [sic] of that activity. The activity is (in a wide sense) empirical and one, it seems, of trial-and-error. Various, what one would in Oslo call: guesses or hypotheses with empirical contents are “tested”. Sometimes the hypothesis is disconfirmed very soon and discarded, or is modified. Sometimes it withstands repeated tests and seems to be firmly established.

The tests consist, roughly, and eulogistically speaking, in asking a number of people well trained in discovering similarities and dissimilarities in conditions about how they use certain phrases or terms.

(Austin & Naess, 1964, p. 148).

According to Naess’s notes, then, the facts about usage gained by consulting suitably qualified people are part of the process of ‘testing’, not of formulating, the hypothesis. It is clear that the summary of the origin of the hypothesis is expressed in words chosen by Naess (‘what one would in Oslo call’); the notion of a ‘guess’ based on some ‘empirical contents’ in fact echoes remarkably closely how Popper was describing the possible process of hypothesis formation in the 1950s. It seems that Naess might then have pressed Austin on some the resemblances between his understanding of his own activity and other contemporary approaches to scientific method. However, Austin’s response seems to have been less than enthusiastic: ‘There is little need felt to place [the methods used at Oxford] within the large framework of the hypothetico-deductive methods’ (Austin & Naess, 1964, p. 149). Austin downplayed the continuity between his work and scientific theory, but Naess’s probing may have identified another of the fundamental difference between them. Like Chomsky, Austin was prepared to ‘go beyond’ the evidence currently available, to form hypotheses and possibly to posit abstract entities. Naess remained committed to accounts that were driven by the data, meaning in turn that the availability of data that could be analysed quantitatively would always be of preeminent importance.

The Oslo meeting may have highlighted some of the main points of difference between Austin and Naess, but it also came near the end of both their work on the philosophy of language. At the time of the meeting Austin had only a few months to live; he died from cancer in February 1960. Naess continued work in Empirical Semantics during the 1960s but his attentions were increasingly directed away from language towards environmental concerns, and he resigned his chair in 1970 to concentrate his efforts on the Deep Ecology movement which he had founded. However, the place of empirical study in philosophy has recently taken centre stage again in relation to the development of experimental philosophy (for some discussion of the role of data and theory in experimental philosophy see Knobe, 2007, p. 82; Weinberg, 2016, p. 20). Moreover, the departure of Naess and Austin more or less coincided with the emergence of various branches of the relatively new discipline of linguistics. The next section is concerned with how the issues relating to the role of experimental data and the role of theory that Austin and Naess discussed in their Oslo meeting have been taken up in linguistics.

## Scientific method in linguistics

Linguistics as it has been practised in the decades since Naess and Austin met in Oslo is a discipline deeply concerned with issues involving data and its relationship to theory. Recorded, intuited, solicited, digitalised and laboratory-collected instances of language use are all current as data in different branches of linguistics (see Chapman, 2008, Chap. 8 for a fuller discussion of these issues). There are also traces to be found of both inductive and hypothetico-deductive scientific methods. Some linguists have addressed them by name. Clift, for instance, while agreeing that ‘pure induction’ is an ‘impossible dream’, argues for the merits of accounts of human linguistic communication which are ‘grounded in the observational’ (Clift, 2005, p. 1642). Others have argued that objective knowledge about language is best derived from a specifically Popperian method of falsification (e.g., Carr, 2009). The picture is complicated, however. Allegiances to a particular type of scientific method are not always explicitly stated, and are not necessarily coextensive with linguistic fields of study.

From the 1960s onwards, many of those who have challenged Chomskyan linguistics have focussed their attentions on collecting and analysing examples of actual language use; sociolinguistics provides a case in point. In a study which would surely have met with Naess’s approval, Labov and his fellow researchers conducted work which relied on carefully designed questionnaires and observations in casual situations to investigate shifts in the pronunciation of two diphthongs taking place over time in the population of Martha’s Vineyard, Massachusetts. Labov proposed that by studying the frequency of different phonetic variants of these diphthongs in different areas of the island ‘it will be possible to reconstruct the recent history of this sound change’ (Labov, 1963, p. 273). He determined what data to collect by conducting ‘exploratory interviews’ in order to identify those features that might be interesting to study (Labov, 1963, p. 279); that is, the project was driven from the start by what the available evidence suggested. Labov was satisfied with the degree of ‘confirmation’ the data gave to his initially tentative claims about ‘the correlation of social patterns with the distributional pattern of one linguistic variable’ (Labov, 1963, p. 308). Coupland, although careful not to classify all twentieth century work in sociolinguistics in the same way, characterises Labov’s method as one of ‘inducing general principles from extensive empirical research’ (Coupland, 2016, p. 3). This seems fair comment, but Labov either saw things differently, or subsequently rethought his approach. A decade after his initial fieldwork was published, admittedly with some caution, he urged linguists to learn from the sciences, where ‘methodology is careful and conscientious search for error in one’s own work, following Karl Popper’s principle that the best theories are the easiest to disconfirm’ (Labov, 1972, p. 99).

The area of linguistics which is perhaps most straightforwardly and explicitly data-driven is conversation analysis. This is a branch of interactional linguistics which developed a little later than sociolinguistics, putting into practice Austin’s ambition of bringing tape recordings of naturally occurring conversations into linguistic study. A group of researchers in Los Angeles led by Harvey Sacks worked in this way for a number of years from the late 1960s onwards, studying the ways in which conversations were structured. In the mid 1970s, they reported that conversation, although spontaneous and unplanned, exhibited much more regular patterning and organisation in terms of turn taking than might have been expected. From the start, Sacks and his team were adamant that anything systematic to be said about conversational behaviour must be based on observation: ‘the existence of orgnaized turn-taking is something that the data of conversation have made increasingly plain’ (Sacks et al., 1974, p. 699). Conversation analysts tend to use qualitative rather than statistical methods, but they have retained this data-first approach. Clift describes the findings of conversational analysis as ‘grounded not in “top-down” assumptions about language use’, but rather ‘embedded in principled, empirical accounts of language use’ (Clift, 2016, pp. 273 and 274). Indeed, conversation analysis is sometimes explicitly identified as an ‘inductive’ field of linguistics (e.g. Hoey & Kendrick, 2017, p. 151).

Both Naess and Austin were aware of the possibilities offered by accruing large quantities of examples of actual linguistic usage, but also of the challenges this would have presented. For Naess, ‘occurrence analysis’ was an ideal in the study of language which could in theory supplant the use of questionnaires, but was in practice unfeasibly difficult. Austin professed not to have ‘time or space’ for such a procedure. The rise in the power and capacity of computing in the decades since both men ceased active engagement in the study of language has of course meant that the analysis of large corpora of naturally occurring texts is now achievable. Corpus linguistics has increased its reach in the past couple of decades, driven by the possibilities of big data analysis, and moving on from a central concern with language itself to focus on more social and ideological issues (O’Keefe and McCarthy, 2022). Corpus linguists celebrate the involvement of non-professionals in their data: ‘[c]orpora are based on naturally occurring texts or spoken languages, which are created everyday by non-expert language users’ (Fellbaum et al., 2004, p. 32).

Many corpus linguists share the enthusiasm of conversation analysts for letting the data guide them. Stubbs, with some apparent caution, confirms that in the case of corpus linguistics: ‘[t]he methods are clearly broadly inductive, in the rough sense that observing large amounts of data leads to the proposal of significant patterns and generalisations’ (Stubbs, 2006, p. 17). Sinclair is careful to emphasise that the corpus linguist is not limited by technology. With an echo of Naess’s reference to the researcher’s ‘eager and open mind’, he argues that: ‘[f]ar from restricting the theorist, the computers will actually encourage hunch-playing and speculation at the creative stage’. He makes clear, however, that the data is to remain firmly in charge: ‘[t]he wealth of data and the ease of access will however encourage the compilation of statements which are firmly compatible with the data’ (Sinclair, 2004, p. 16). A very recent overview of corpus linguistics argues that ‘[t]he persuasiveness of our arguments about language depends on the plausible and robust interpretation of the principled empirical evidence which the data throw up’ (O’Keeffe and McCarthy, 2022, p. 6).

In a recent example of a corpus-based and data-driven study, Dong et al. investigate the different distribution of linguistic markers of attitude in texts about the 2020 pandemic in the COVID-19 academic corpus and the Coronavirus Corpus of media reports. Attitude is manifested linguistically by expressions of affect (words expressing emotional evaluation, such as ‘interesting’, ‘sad’, ‘surprising’), of judgement (words expressing ethical evaluation of behaviour, such as ‘reasonable’, ‘logical’, ‘right’) and appreciation (words expressing value-laden evaluation, such as ‘important’, ‘significant’, ‘useful’). The aim of the study is ‘to uncover the developmental patterns in the semiotic system of attitudinal construction’ in the two genres and the research questions are open and exploratory (e.g., ‘What cross-corpora differences can be found in the attitudinal response taken towards COVID-19 in the academic and media corpus?’, Dong et al., 2021, p. 534). Their findings are driven by the statistically analysed data. For instance, ‘the media corpus used significantly more appreciation-oriented expressions that the academic corpus’ while the academic corpus had a higher preponderance of judgement, showing a greater reliance on expressions of attitude orientated towards logic (Dong et al., 2021, p. 543).

Technological advances have not uniformly pointed researchers in linguistics to data-driven or inductive studies, however. Recent developments in semantics and pragmatics offer an interesting final case study of the role of theory, and indeed of the question of what is to count as ‘experimental’ data, in linguistics. The tradition in pragmatics which developed from ordinary language philosophy has always included a theoretical element: an impetus, to borrow Chomsky’s phrase, to ‘go beyond the evidence’. As discussed in Sect. 3 above, speech act theory abstracts away from individual utterances to posit the ‘locutionary’, ‘illocutionary’ and ‘perlocutionary’ acts. Gricean pragmatics, too, has been premised on the existence of something not immediately available to observation (a distinction between ‘what is said’ and ‘what is implicated’, Grice, 1975, p. 24) in order to explain what can be observed (the ways in which forms of word can communicate different messages in different contexts). Grice drew attention to the fact that in postulating the cooperative principle and its associated maxims he was purposefully going beyond ‘empirical fact’ in an attempt ‘to find a basis that underlies these facts’ (Grice, 1975, p. 29). This century has seen rapid growth in the use of experimental methods in pragmatics, with researchers generally engaged in testing and perhaps comparing existing pragmatic theories. For instance, Breheny identifies Levison’s account of default implicatures as a ‘testable proposal about the language-pragmatics interface’ (Breheny, 2019, p. 46). If Levinson is correct that what are sometimes described as ‘scalar implicatures’ arise by default unless supressed by strong contextual clues, then it would seem to follow that response tasks which required participants to access scalar implicatures should be successfully completed ‘either faster or at least not slower than’ those which do not (Breheny, 2019, p. 47). Breheny’s survey of the data from various studies points to the ‘disconfirmation’ of Levinson’s claim.

Experimental pragmatics is perhaps the branch of present day linguistics in which explicit statements about scientific method are most frequent. Gibbs has written extensively on metaphors, and subjected Grice’s account of them to experimental testing. In so doing, he has looked for specific predictions that Grice’s theory might be taken to make, since: ‘I strongly embrace the belief that the best ideas in linguistic-pragmatics are those that can be experimentally examined and potentially falsified (where failing to falsify allows one to claim scientific evidence in support of a hypothesis)’ (Gibbs, 2004, p. 69). Other researchers have identified the need for a pragmatic theory not just to make predictions about observable data, but to do so in ways which distinguishes it from other, potentially competing theories, since experimental evidence compatible with the predictions of a particular pragmatic theory but also with those of another pragmatic theory ‘provides only weak support’ for the theory under investigation’ (Van der Henst and Sperber, 2004, p. 141). It is this very closeness between the apparent predictions of different pragmatic theories which, for Cummins and Katsos, make experimental data crucial. In a comment that would not have pleased Austin they argue that: ‘[t]he cluster of predictions that can differentiate between competing accounts is too fine-grained to be reliably adjudicated by the traditional tools of the armchair linguist, reflective introspection and intuition’ (Cummins and Katsos, 2019, p. 3).

However, in present-day pragmatics and semantics there is some support to be found for Austin’s alternative account of ‘experimental data’ in the study of language. Acknowledging an observation from an anonymous pre-publication reader, Cummins and Katsos allow that: ‘some may consider introspective research and one-on-one elicitation as (mini-) experiments, with few items, few speakers (the researcher and her colleagues), and few or no filters’ (Cummins and Katsos, 2019, p. 4). In a challenge to the dominance of laboratory methods in semantics, Jacobson argues that ‘the traditional use of just one or a few informants is every bit as experimental’ (Jacobson, 2018, p. 48). Jacobson goes further than Cummins and Katsos’s anonymous reader and argues that reverence for data collected from numerous non-philosophical informants can in fact have a negative effect. Austin warned Naess of the dangers of ‘uncritical expectations that the use of the techniques in new fields will give better results than non-formal, intuitional, ways’. Similarly, Jacobson cautions that ‘the implicit or explicit claims that the traditional methodology is “unscientific” and that all work stemming from this methodology therefore cannot be trusted could be quite damaging to the enterprise of semantics’ (ibid). For Jacobson, there are positive advantages to be found in researchers providing their own experimental data.

We know that if the sentence *Mitka killed Porky* is true in some situation, then it must be that Porky died. If a subject tells us otherwise, it means they have a different meaning for some of the lexical items, they are using *kill* figuratively, they do not speak English, they take the proper name *Porky* to refer to someone other than the grunting pig that we have in mind, or they simply didn’t understand the task. No one would revise their semantic theory on the basis of judgments like this.

(Jacobson, 2018, p. 60)

There is nothing dismissive in Jacobson’s attitude towards non specialist informants, but she does here echo Austin’s contention in Oslo that non experts might, for whatever reason, ‘give wrong answers’ and therefore that what the researcher already knows about the language may be a more valuable source of data.

## Conclusion

Arne Naess and J. L. Austin were both leading proponents of empiricism in the philosophy of language in the mid-twentieth century but, as the record of their meeting in Oslo in 1959 exemplifies, they disagreed on what this meant for philosophical practice. Very broadly, Naess saw accumulated evidence about linguistic usage as of primary importance and argued that the quantities of data necessary for such study must be collected from the behaviour and the judgements of non-philosophical informants. Austin afforded more room in his philosophy of language for theoretical constructs which were explanatory but which might go beyond what could be observed. In principle, although not in his own practice, he acknowledged that the linguistic behaviour of non-experts could provide relevant data to the study of language, but he was wary of judgements about usage collected from those without suitable training. He maintained that the smaller amounts of data necessary to his approach were most reliably to be drawn from the expert knowledge of language use of researchers themselves, and at times he argued that such data could justifiably be labelled ‘experimental’. These differing opinions find resonances in the empirical study of language which followed after Naess and Austin’s work, and carries on into the present day, in various branches of linguistics. The divergent attitudes to scientific method, in terms both of the role of theory and of the nature of properly ‘experimental’ data expressed by Austin and Naess in their 1959 Oslo conversation continue to shape the ways in which we understand and explain human language.

## Footnotes

1 Tape, 29 January 1983, Grice in conversation with Richard Warner and Judith Baker, H.P.Grice Papers, BANC MSS 90/135 c, The Bancroft Library, University of California, Berkeley.
